# Imaging Immune Checkpoint Inhibitor Myocarditis: Can We Do Better?

**DOI:** 10.1016/j.jaccao.2025.08.001

**Published:** 2025-09-11

**Authors:** Edward Doris, Charlotte Manisty, Sivatharshini Ramalingam, Gary J.R. Cook, Alexander R. Lyon, Muhummad Sohaib Nazir

**Affiliations:** a School of Biomedical Engineering and Imaging Sciences, King’s College London, Guy’s and St. Thomas’ Hospital, London, United Kingdom; b Cardio-Oncology Service and Cardio-Oncology Centre of Excellence, Royal Brompton Hospital, London, United Kingdom; c Institute of Cardiovascular Science, University College London, London, United Kingdom; d Barts Heart Centre, Barts Health NHS Trust, London, United Kingdom; e King’s College London and Guy’s and St. Thomas’ PET Centre, School of Biomedical Engineering and Imaging Sciences, King’s College London, London, United Kingdom

**Keywords:** biomarkers, cardiac magnetic resonance, cardiomyopathy, guidelines, immunotherapy, myocarditis, nuclear imaging

Immune checkpoint inhibitors (ICIs) have markedly improved clinical outcomes for cancer patients. By targeting pathways involved in immune cell modulation, they allow the immune system to overcome maneuvers used by malignant cells to avoid detection. Despite being highly effective cancer treatments, they are not without risk. Through an immune mechanism from activated T cells, immune-related adverse events can target any organ, including the cardiovascular system. ICI myocarditis is an important but rare complication with a high fatality rate in severe cases, whereas milder cases are increasingly diagnosed and present management challenges. As such, there is a clinical urgency for accurate and timely diagnosis. However, accurate diagnosis and risk stratification of ICI myocarditis can be challenging, with important limitations to currently available diagnostic methods.

Endomyocardial biopsy is considered the gold standard for the diagnosis of ICI myocarditis but has several limitations. First, because of the heterogeneous nature of myocardial inflammation,^1^ it is possible to biopsy noninflamed myocardium, leading to false negatives. Additionally, standard criteria used in histopathologic diagnosis of myocarditis are relatively insensitive to ICI myocarditis.^2^ Endomyocardial biopsy is an invasive test with a small risk for serious side effects, and there is often a clinically meaningful delay from suspicion of diagnosis, obtaining biopsy, and receiving diagnosis. This clearly implicates the need for a rapid, noninvasive alternative to inform timely management and reduce mortality.

The clinical diagnosis of ICI myocarditis is obtained from a rise in serum troponin associated with either diagnostic features of myocarditis on cardiac magnetic resonance imaging (CMR) or minor criteria including clinical symptoms and electrocardiographic abnormalities according to European Society of Cardiology (ESC) guidelines.^3^ Guidelines recommend echocardiography for assessment of myocarditis; although insufficient for diagnosis, it may provide important supportive information such as left ventricular ejection fraction, presence of pericardial effusion, and exclusion of other conditions. Guidelines recognize that CMR features specific to ICI myocarditis are not well described. The modified Lake Louise criteria are recommended but were developed for the diagnosis of myocarditis in general and are based on presence of myocardial edema by T2 mapping or T2-weighted images and nonischemic myocardial injury through T1 mapping or extracellular volume values or late gadolinium enhancement (LGE). However, the criteria are not based on studies in ICI myocarditis, and thus the diagnostic performance in this cohort is unknown. Additionally, recent evidence demonstrates that CMR often does not demonstrate typical imaging findings found in other forms of myocarditis, depending on the timing of the scan. A cohort study of patients with biopsy-proven ICI myocarditis and CMR demonstrated that LGE was present in only 39% of cases and increased T2 short-tau inversion recovery (T2 STIR) in only 25%.^4^ Additionally, 48% of this cohort had neither positive T2 STIR nor LGE. In a multicenter registry of 136 patients with ICI myocarditis based on endomyocardial biopsy or ESC criteria for myocarditis, T1 mapping was found to have a greater diagnostic accuracy compared with T2 mapping for the detection of myocarditis, although an elevated T1 can be nonspecific in context of hypertension, aortic stenosis, and cardiac amyloidosis.^5^ There were no major adverse cardiovascular events in patients with normal T1 values, highlighting the potential value of T1 mapping in CMR for risk stratification and potential to monitor treatment response. In summary, there is a lack of robust CMR features specific for the diagnosis of ICI myocarditis, likely reflective of the unclear mechanism behind its development.

Positron emission tomography (PET) has potential for the diagnosis of ICI myocarditis. American Society of Nuclear Cardiology guidelines recommend ^18^F-fluorodeoxyglucose (FDG) PET for the diagnosis of inflammatory conditions such as cardiac sarcoidosis. ESC cardio-oncology guidelines do not explicitly recommend PET when CMR is not available, although its low sensitivity is acknowledged.^3^ Indeed, the evidence on the use of ^18^F-FDG PET in the diagnosis of myocarditis is limited to a single study of patients (n = 60) referred for suspected ICI myocarditis that demonstrated sensitivity of only 9.5% in patients with biopsy-proven myocarditis.^6^ However, it is not clear whether patients adhered to strict glucose fasting to avoid normal physiological ^18^F-FDG uptake, and many patients were on steroid therapy, and thus the findings need careful interpretation. Furthermore, ^18^F-FDG PET relies on the detection of activity that exhibits increased glucose uptake, such as from activated macrophage activity, but this may not necessarily be the pathologic mechanism in ICI myocarditis.

There are several reasons why imaging in the diagnosis of ICI myocarditis may be problematic. One limitation of imaging techniques lies with the timing with which they are performed. Patients with suspected ICI myocarditis are often commenced on immunosuppressive treatment before imaging is performed, which may suppress imaging-derived inflammatory signal and lead to lower diagnostic yield. Similarly, if imaging is performed too early, typical hallmark signs may not yet be apparent.^4^ ^18^F-FDG PET can be pragmatically difficult to perform because of limited availability of PET centers and restrictive dietary requirements. To suppress background myocardial glucose use, American Society of Nuclear Cardiology guidelines recommend adherence to a strict carbohydrate-free diet for 24 hours, followed by 12 hours of fasting. Even when this regimen is followed, there is a chance of incomplete suppression and nondiagnostic images. Finally, currently available imaging techniques suffer from a lack of specificity. Because of a lack of knowledge surrounding the pathophysiology implicated in ICI myocarditis, there have been no mechanisms identified unique to the disease process that provide a specific imaging approach.

Additional diagnostic difficulties arise in the differentiation of ICI myocarditis and acute coronary syndrome, as ICI is known to accelerate atherosclerosis. Being able to distinguish the 2 entities to choose the appropriate therapy in a timely fashion remains an important gap in our diagnostic pathways.

Given the unique positioning of molecular imaging in its ability to image specific biological pathways depending on the radiotracer used, there is strong potential for a clinically useful role in the future. Further work is required to understand the sequences of molecular changes that occur in ICI myocarditis, which may in turn allow molecular imaging agents to target specific points in the myocarditis process.

There are several possible avenues that, in the future, may improve our ability to efficiently and expediently diagnose ICI myocarditis. One possibility is through the use of hybrid PET and magnetic resonance imaging, which can provide complementary information in myocarditis; myocardial edema and injury can be detected using CMR and ^18^F-FDG PET for glucose use as a surrogate of myocardial inflammation. This may improve diagnostic yield or alternatively allow us to effectively “rule out” in a single scan. There has been one study of PET and magnetic resonance imaging to assess concordance, but more research is needed, with comparison with the current diagnostic pathway and specifically with endomyocardial biopsy.^7^

PET also holds much promise in improving diagnostic pathways through the development of targeted radiotracers that may be able to more effectively target pathways involved in ICI myocarditis. For example, programmed cell death 1 is involved in the modulation of T cell receptor–mediated signaling, and alongside its ligand counterpart programmed cell death ligand 1 (PD-L1) is a target for several ICIs. PD-L1 expression is up-regulated in the myocardium of patients with ICI myocarditis, which could be a target for molecular imaging. A novel radiotracer targeting PD-L1 has been developed that may allow the quantification of myocardial PD-L1 expression,^8^ though this requires further investigation in prospective clinical trials to determine its role in predicting risk for developing myocarditis from ICI therapy.

A murine study has implicated CD8^+^ T cells as having a significant role in the pathophysiology of ICI myocarditis.^9^ The ability to quantify CD8 activity during the acute phase of ICI myocarditis may generate specific imaging readouts of the inflammatory phase of ICI myocarditis and have been used in preclinical and early phase clinical trials. This has implications not only in the diagnosis of ICI myocarditis but also in risk stratification for ICI myocarditis and surveillance for chemotherapy.^10^ More broadly, novel PET tracers have been developed for the assessment of inflammation, with promising early data in preclinical studies such as with CD45 PET and folate receptor β although their precise role in the assessment of ICI myocarditis warrants further investigation.

Fibroblast activation protein inhibitor (FAPI) PET may have utility in ICI myocarditis. Fibroblast activation protein is highly expressed on cancer-associated fibroblasts and as such is used as a target for cancer imaging via the use of the FAPI. A cohort study of patients with definite ICI myocarditis demonstrated increased ^68^Ga-labeled FAPI uptake on hybrid PET/computed tomography compared with patients treated with ICIs without myocarditis;^1^ importantly, in those patients who underwent CMR, findings did not fulfill the modified Lake Louise criteria for myocarditis diagnosis. Although limited by its sample size, this study suggests potential promise for FAPI PET for diagnosis of ICI myocarditis.

The radiotracer ^68^Ga-DOTATOC shows potential in diagnosis of ICI myocarditis in one study that demonstrated pathologic cardiac uptake in patients with clinical suspected myocarditis without associated CMR abnormalities.^11^ Furthermore, patients with myositis also demonstrated ^68^Ga-DOTATOC uptake in skeletal muscle, suggesting that this radiotracer may also provide an imaging readout of the systemic complications from ICI such as with myositis.

Complementary to the development of radiotracers is the technological advancement of long-axis field of view (LAFOV) PET. Increasing the number of detectors to cover a longer axial view of the human body allows a marked increased sensitivity and high temporal resolution dynamic imaging. The combination of this new technology with novel radiotracers may allow the detection of pathology that was not possible previously. Although the expense and limited availability of LAFOV PET limits its use, the development of more cost-efficient materials to bring down cost means that this could soon be a viable option for clinical use.

Early recognition of deleterious adverse effects is critical with the growing role of ICIs as effective cancer therapies. Current imaging techniques, though useful, have important limitations in terms of sensitivity and specificity and are hampered by the need to initiate early immunosuppressive treatment. More work needs to be undertaken to understand the temporal changes that occur in myocarditis that may in turn guide clinicians to the optimal timing of appropriate imaging of this complex diagnosis. Novel imaging techniques with hybrid imaging, LAFOV PET, and specific radiotracers for ICI myocarditis show great promise, and further research in prospective multicenter clinical studies is needed to allow us to translate these nascent technologies into clinical practice for improved diagnosis for patients with suspected ICI myocarditis.

## Funding Support and Author Disclosures

The authors acknowledge financial support from the Department of Health through the National Institute for Health and Care Research (NIHR) MedTech Co-Operative for Cardiovascular Disease at Guy’s and St. Thomas’ NHS Foundation Trust. The Royal Brompton Cardio-Oncology Centre for Excellence is supported by the Big Heart Foundation. Dr Doris is funded by the Rosetree’s Trust. Dr Manisty is supported by the NIHR Biomedical Research Centre at University College London Hospitals NHS Trust. Dr Lyon is supported by the Fondation Leducq Network of Excellence in Cardio-Oncology. The views expressed are those of the authors and not necessarily those of the National Health Service or the NIHR. Dr Manisty has received speaker, advisory board, or consultancy fees from Beigene, AstraZeneca, Pfizer, Abbott Medical, and Biotronik; and is a board member of MycardiumAI. Dr Cook has received consultancy fees and research support from NanoMab Technology. Dr Lyon has received speaker, advisory board, or consultancy fees and/or research grants from Janssen-Cilag, Astellas Pharma, Pfizer, Novartis, Servier, AstraZeneca, Bristol Myers Squibb, GlaxoSmithKline, Amgen, Takeda, Roche, Clinigen Group, Eli Lily, Eisai, Ferring Pharmaceuticals, Boehringer Ingelheim, Akcea Therapeutics, Myocardial Solutions, iOWNA Health, Heartfelt Technologies, ARMA Biosciences, and EPICLIFE. Dr Ramalingam has received speaker or advisory board fees and research grants from Ferring, Accord, AstraZeneca, Novo Nordisk, and Novartis. Dr Nazir has received speaker, advisory board, or consultancy fees and/or research grants from Beigene, Johnson & Johnson, and Myocardial Solutions. Dr Doris has reported that he has no relationships relevant to the contents of this paper to disclose.

## References

[1] Finke D., Heckmann M.B., Herpel E. (2021). Early detection of checkpoint inhibitor-associated myocarditis using ^68^Ga-FAPI PET/CT. Front Cardiovasc Med.

[2] Jimenez J., Kostelecky N., Mitchell J.D. (2023). Clinicopathological classification of immune checkpoint inhibitor-associated myocarditis: possible refinement by measuring macrophage abundance. Cardiooncology.

[3] Lyon A.R., Lopez-Fernandez T., Couch L.S. (2022). 2022 ESC guidelines on cardio-oncology developed in collaboration with the European Hematology Association (EHA), the European Society for Therapeutic Radiology and Oncology (ESTRO) and the International Cardio-Oncology Society (IC-OS). Eur Heart J.

[4] Zhang L., Awadalla M., Mahmood S.S. (2020). Cardiovascular magnetic resonance in immune checkpoint inhibitor-associated myocarditis. Eur Heart J.

[5] Thavendiranathan P., Zhang L., Zafar A. (2021). Myocardial T1 and T2 mapping by magnetic resonance in patients with immune checkpoint inhibitor-associated myocarditis. J Am Coll Cardiol.

[6] Ederhy S., Devos P., Pinna B. (2022). ^18^F-fluorodeoxyglucose positron emission tomography/computed tomography imaging for the diagnosis of immune checkpoint inhibitor-associated myocarditis. Arch Cardiovasc Dis.

[7] Tong J., Vogiatzakis N., Andres M.S. (2024). F-FDG positron emission tomography imaging in suspected immune checkpoint inhibitor myocarditis. Cardiooncology.

[8] Nazir M.S., Hughes D.J., Chand G. (2023). [^99m^Tc]-labelled anti-programmed death-ligand 1 single-domain antibody SPECT/CT: a novel imaging biomarker for myocardial PD-L1 expression. EJNMMI Res.

[9] Axelrod M.L., Meijers W.C., Screever E.M. (2022). T cells specific for alpha-myosin drive immunotherapy-related myocarditis. Nature.

[10] Zhang J., Du B., Wang Y. (2024). The role of CD8 PET imaging in guiding cancer immunotherapy. Front Immunol.

[11] Boughdad S., Latifyan S., Fenwick C. (2021). ^68^Ga-DOTATOC PET/CT to detect immune checkpoint inhibitor-related myocarditis. J Immunother Cancer.

